# Side differences in upper quarter mobility/stability are not related to serve velocity in tennis players with different levels of training experience

**DOI:** 10.1186/s13104-024-06944-z

**Published:** 2024-09-26

**Authors:** Johanna Lambrich, Stefan Panzer, Thomas Muehlbauer

**Affiliations:** 1 Division of Movement and Training Sciences, Biomechanics of Sport, University of Duisburg-Essen, Gladbecker Str. 182, 45141 Essen, Germany; 2 Institute of Sport Science, Saarland University, Saarbrücken, Germany

**Keywords:** Racket sport, Serve style, Athletes, Y Balance Test-Upper Quarter, Inter-limb asymmetry

## Abstract

**Objective:**

Tennis is characterised by repetitive serves and strokes predominately performed with one arm. This can lead to differences in upper quarter mobility/stability between the stroke and non-stroke arm, which could even enlarge with increasing training experience and negatively affect serve velocity. Thus, we determined side differences (i.e., limb symmetry index) in upper quarter mobility/stability and their association with flat and slice serve velocity in advanced (ITN ≤ 4) female and male tennis players (*N* = 42, mean age = 23.9 ± 9.3 years) with different levels of training experience (< 2 years: *n* = 14, 2–5 years: *n* = 17, 6–8 years: *n* = 11).

**Results:**

Y Balance Test-Upper Quarter (YBT-UQ) side difference (i.e., composite score) and performances (i.e., medial reach) were largest in players with the lowest level of training experience (i.e., < 2 years). Further, YBT-UQ performances (i.e., medial reach and composite score) but not side differences were significantly correlated with flat and slice serve velocity, particularly in less experienced players. Our results suggest that significant side differences in upper quarter mobility/stability occur in less experienced players (indicative of increased injury risk) but they are not related to tennis-specific performance (i.e., serve velocity).

**Supplementary Information:**

The online version contains supplementary material available at 10.1186/s13104-024-06944-z.

## Introduction

Tennis is characterised by mainly unilateral hitting actions (e.g., serve, groundstroke, etc.). This may result in side differences (i.e., inter-limb asymmetry) between the stroke arm and non-stroke arm in terms of upper limbs’ performance, which can further increase with years of training. This experience-related change has been interpreted as ‘functional specialisation’ [1], whereby structural and functional adaptations in the neuromuscular system occur because of long-term motor practice. Even if the appearance of a functional specialisation initially suggests an advantage (e.g., hard and fast hitting actions), there is evidence [2] for the occurrence of physical performance decrements if asymmetry increases above a certain level (~ 10%). For example, Gonzalo-Skok et al. [3] reported that neuromuscular asymmetry was associated with inferior physical performance (i.e., lower vertical jump height) in young elite athletes. Moreover, Madruga-Parera et al. [4] investigated elite youth tennis players and found significant side differences in the lower limbs for measures of muscle power, change of direction speed, and balance.

Despite the evidence of negative effects of neuromuscular asymmetry on athletic performance of the lower limbs, studies about the influence on upper limb performance are lacking. This is surprising, since neuromuscular asymmetry in upper quarter mobility/stability has been previously associated with an increased risk (i.e., risk ratio: 1.2) for a future time-loss musculoskeletal injury [5]. Therefore, the objectives of the present study were to assess side differences between the stroke arm and non-stroke arm in upper quarter mobility/stability and their association with serve velocity in tennis players with different levels of training experience. We hypothesised that due to the tennis-specific unilateral hitting actions side differences in upper quarter mobility/stability between arms would be present, increase with advancing level of training experience, and are negatively associated with serve velocity.

## Main text

### Methods

#### Participants

Forty-two advanced (ITN ≤ 4) tennis players who were free of any musculoskeletal dysfunction, neurological impairment, or orthopaedic pathology participated in this study (Table 1). In accordance with Swann et al. [6], players were divided into three groups differing in the level of training experience (< 2 years: *n* = 14, 2–5 years: *n* = 17, 6–8 years: *n* = 11).

**Table 1:** Characteristics of the tennis players by level of training experience

Characteristic	All players (*N* = 42)	YoT < 2 (*n* = 14)	YoT 2–5 (*n* = 17)	YoT 6–8 (*n* = 11)	*p*-value (ƞ_*p*_^2^)
Sex [f/m]	16/24	3/11	5/12	8/3	–
Age [years]	23.9 ± 9.3	22.7 ± 10.0	23.9 ± 11.0	25.5 ± 4.7	0.772 (0.01)
Body height [cm]	177.6 ± 8.5	180.2 ± 7.6	176.6 ± 9.9	176.0 ± 6.9	0.383 (0.05)
Body mass [kg]	71.4 ± 11.1	71.8 ± 10.8	72.7 ± 12.4	68.5 ± 9.8	0.622 (0.02)
Body mass index [kg/m^2^]	22.5 ± 2.4	22.1 ± 2.6	23.1 ± 2.5	22.0 ± 2.1	0.371 (0.05)
Stroke arm [l/r]	3/39	0/14	0/17	3/8	–
Stroke arm length [cm]	90.6 ± 4.3	92.1 ± 3.6	89.9 ± 4.6	90.0 ± 4.5	0.242 (0.07)
Non-stroke arm length [cm]	89.4 ± 4.5	91.1 ± 3.8	88.7 ± 4.5	88.5 ± 5.1	0.015 (0.19)

Values are expressed as mean ± standard deviation. Figures in brackets are effect sizes (*η*_p_^2^) with 0.02 ≤ *η*_p_^2^ ≤ 0.12 indicating small, 0.13 ≤ *η*_p_^2^ ≤ 0.25 indicating medium, and *η*_p_^2^ ≥ 0.26 indicating large effects. f: female; l: left; m: male, r: right, YoT: years of training

#### Testing procedures

After entering the outdoor clay court, the players received verbal information about the testing procedure and a live demonstration of both serve types (i.e., flat and slice) performed by the experimenter. Thereafter, their body mass, body height, and arm length were measured followed by a 10-minutes standardised warm-up including speed, agility, and stretching exercises as well as 10 serves at submaximal speed. Subsequently, each player was required to execute flat and slice serves at maximum speed in a randomised order until a total of five serves of each type successfully reached the service box. Right-handed players performed the serves from the deuce side, while left-handed players served from the advantage side. Every player used their own racket and new balls. The order of both serve types was randomised between players. Lastly, the upper quarter mobility/stability was assessed.

#### Assessment of anthropometric variables

Body mass was measured with an electronic scale (Seca 803, Basel, Switzerland) to the nearest 100 g and body height was measured using a stadiometer (Seca 217, Basel, Switzerland) to the nearest 0.1 cm, with participants wearing light clothing, standing straight and upright without shoes. Arm length (to the nearest 0.1 cm) was measured from the distal tip of the middle finger, with the shoulder at 90-degree abduction, to the seventh cervical spinous process using a measuring tape [7].

#### Assessment of serve velocity

Ball speed was assessed using a “Stalker Pro” Doppler radar gun (working frequency: 35.1 GHz, measuring range: 0–400 km/h, accuracy: 0.16 km/h; Applied Concepts Inc., Plano, TX, USA). The radar gun was positioned on the centre of the baseline, 4.0 m behind the player, aligned at a height of ~ 2.2 m (approximate height of ball contact) and pointing down to the centre of the court [8]. The achieved ball speed was verbally provided after each serve and the fastest serve (km/h) for each type was used for subsequent analysis. Reliability of the radar gun has been shown in a previous study [9].

#### Assessment of upper quarter mobility/stability

Upper quarter mobility/stability was assessed using the Y Balance Test-Upper Quarter (YBT-UQ). While maintaining a single arm push up position on the central platform of the test kit (Move2Perform, Evansville, IN, United States), participants were asked to push the reach indicator along three pipes with the contralateral arm in the following order: (1) medial (MD), (2) inferolateral (IL), and (3) superolateral (SL) reach direction (see Additional file 1). Players performed three trials for each arm separated by 30 s and the greatest reach distance (cm) in each direction was used for subsequent analysis. A trial was discarded and repeated if the player (1) failed to maintain the single arm push up position (i.e., touched the floor with the reach arm), (2) failed to remain in contact with the reach indicator at the most distal point (i.e., pushed the reach indicator to achieve greater distance), (3) used the reach indicator to support weight (i.e., mechanical support), or (4) failed to return the reach arm to the centre of the test kit. Reach distance was normalised to arm length (i.e., (reach distance / arm length) * 100)) and the composite score was calculated (i.e., [(MD + IL + SL) / (3 * arm length)] * 100) [10]. Lastly, the limb symmetry index (LSI) was calculated as the mean score for the non-stroke arm divided by the mean score for the stroke arm, and then multiplied by 100 [11].

#### Statistical analysis

Data were analysed using JASP version 0.16.4.0 (Amsterdam, The Netherlands) and are presented as mean ± standard deviation. After normal distribution (Shapiro-Wilk test) and homogeneity of within variance/sphericity (Levene test) was confirmed, a univariate ANOVA was performed to detect between-group differences. Bonferroni-adjusted post-hoc analyses were performed if a significant difference occurred. Partial eta-squared (*η*_p_^2^) was calculated and reported as small (0.02 ≤ *η*_p_^2^ ≤ 0.12), medium (.13*η*_p_^2^ ≤ 0.25), or large (*η*_p_^2^ ≥ 0.26) for the ANOVA and Cohen’s *d* was determined and interpreted as trivial (0 ≤ *d* ≤ 0.19), small (0.20 ≤ *d* ≤ 0.49), moderate (0.50 ≤ *d* ≤ 0.79), or large (*d* ≥ 0.80) for the post-hoc analyses. Further, associations between YBT-UQ side difference and performance with serve velocity were separately calculated for each serve type using Pearson’s product moment correlation coefficient. Coefficients were interpreted as weak (*r* = .10–0.35), moderate (*r* = .36–0.67), or strong (*r* = .68–1.00) [12]. The alpha value was a priori set at *p* < .05 for all analyses.

## Results

The ANOVA showed small to medium group discrepancies in YBT-UQ side difference, that were significant for the composite score (LSI: *p* = .012, *η*_p_^2^ = 0.20) only (Table 2). Post-hoc analyses yielded a large-sized difference between means (*p* = .009, *d* = 1.26), with the group with the lowest training experience (YoT: <2) showing higher values compared to those with the highest (YoT: 6–8) experience level. Further, we detected small to medium group differences in YBT-UQ performance, that reached the level of significance for the medial reach direction (stroke arm: *p* = .040, *η*_p_^2^ = 0.15; non-stroke arm: *p* = .037, *η*_p_^2^ = 0.16) only. Again, post-hoc analyses revealed large-sized differences between means (stroke arm: *p* = .031, *d* = 1.1; non-stroke arm: *p* = .029, *d* = 1.1), with players with the lowest training experience (YoT: <2) achieving higher values compared to those with the highest (YoT: 6–8) experience level. Concerning tennis-specific performance, flat and slice serve velocity did not significantly differ between groups.

**Table 2:** Y Balance Test-Upper Quarter (YBT-UQ) side differences and performance and tennis-specific performance by level of training experience

Outcome	YoT: <2 (*n* = 14)	YoT: 2–5 (*n* = 17)	YoT: 6–8 (*n* = 11)	*p*-value (ƞ_*p*_^2^)
**YBT-UQ: side difference**				
LSI for the medial reach [% AL]	4.7 ± 3.3	3.8 ± 2.8	3.9 ± 2.8	0.700 (0.02)
LSI for the inferolateral reach [% AL]	11.5 ± 8.1	9.4 ± 7.7	5.3 ± 3.2	0.105 (0.11)
LSI for the superolateral reach [% AL]	9.4 ± 7.8	7.5 ± 4.9	6.3 ± 5.3	0.437 (0.04)
LSI for the composite score [% AL]	6.6 ± 4.3	4.3 ± 2.9	2.5 ± 1.9	0.012 (0.20)
**YBT-UQ: performance**				
SA: medial reach [% AL]	115.5 ± 10.2	111.6 ± 8.9	105.3 ± 9.9	0.040 (0.15)
SA: inferolateral reach [% AL]	100.7 ± 12.1	106.2 ± 10.9	99.4 ± 11.3	0.245 (0.07)
SA: superolateral reach [% AL]	80.0 ± 10.7	76.8 ± 10.0	76.8 ± 11.1	0.663 (0.02)
SA: composite score [% AL]	98.7 ± 8.5	98.2 ± 6.0	93.8 ± 10.0	0.272 (0.07)
NSA: medial reach [% AL]	116.3 ± 8.9	110.7 ± 8.5	105.8 ± 12.3	0.037 (0.16)
NSA: inferolateral reach [% AL]	105.0 ± 10.8	108.7 ± 10.3	101.4 ± 13.3	0.257 (0.07)
NSA: superolateral reach [% AL]	78.2 ± 12.3	75.2 ± 12.3	77.8 ± 12.2	0.770 (0.01)
NSA: composite score [% AL]	99.8 ± 7.7	98.2 ± 6.6	95.0 ± 10.6	0.348 (0.05)
**Tennis-specific performance**				
Flat serve velocity [km/h]	160.4 ± 18.9	158.6 ± 16.7	150.8 ± 15.3	0.357 (0.05)
Slice serve velocity [km/h]	145.1 ± 19.0	141.5 ± 16.1	129.4 ± 15.8	0.071 (0.13)

Values are expressed as mean ± standard deviation. Figures in brackets are effect sizes (*η*_p_^2^) with 0.02 ≤ *η*_p_^2^ ≤ 0.12 indicating small, 0.13 ≤ *η*_p_^2^ ≤ 0.25 indicating medium, and *η*_p_^2^ ≥ 0.26 indicating large effects. AL: arm length, LSI: limb symmetry index, NSA: Non-stroke arm; SA: stroke arm; YBT-UQ: Y Balance Test-Upper Quarter; YoT: years of training

Non-significant weak to moderate positive and negative correlations were observed between YBT-UQ reach asymmetry and flat/slice serve velocity, regardless of training experience (Table 3). However, YBT-UQ reach performance (i.e., medial direction) was significantly positively correlated (*r* = .36 to 0.65, *p* < .05 to 0.001) with flat and slice serve velocity in all players and in those with YoT: <2 and YoT: 2–5.

**Table 3:** Correlations of Y Balance Test-Upper Quarter side differences and performance with serve velocity by level of training experience

Outcome	Flat serve velocity [km/h]				Slice serve velocity [km/h]			
	All players (*N* = 42)	YoT: <2 (*n* = 14)	YoT: 2–5 (*n* = 17)	YoT: 6–8 (*n* = 11)	All players (*N* = 42)	YoT: <2 (*n* = 14)	YoT: 2–5 (*n* = 17)	YoT: 6–8 (*n* = 11)
**YBT-UQ: side difference**								
LSI for the medial reach [% AL]	0.14	− 0.26	0.39	0.43	0.04	− 0.39	0.30	0.31
LSI for the inferolateral reach [% AL]	− 0.15	− 0.48	− 0.15	0.22	− 0.05	− 0.23	− 0.20	− 0.06
LSI for the superolateral reach [% AL]	0.08	0.12	− 0.15	0.19	0.28	0.31	0.01	0.42
LSI for the composite score [% AL]	− 0.05	− 0.32	− 0.09	0.20	0.12	− 0.14	0.06	0.07
**YBT-UQ: performance**								
SA: medial reach [% AL]	0.38*	0.48	0.32	0.11	0.57***	0.57*	0.54*	0.35
SA: inferolateral reach [% AL]	− 0.01	0.18	− 0.13	− 0.24	0.14	0.18	0.07	0.07
SA: superolateral reach [% AL]	0.10	0.43	− 0.06	− 0.21	0.12	0.28	− 0.05	0.04
SA: composite score [% AL]	0.20	0.46	0.05	− 0.13	0.36*	0.43	0.29	0.16
NSA: medial reach [% AL]	0.41**	0.65*	0.26	0.20	0.55***	0.53*	0.47	0.49
NSA: inferolateral reach [% AL]	− 0.10	− 0.09	− 0.10	− 0.33	− 0.02	− 0.03	− 0.12	− 0.15
NSA: superolateral reach [% AL]	0.10	0.35	− 0.07	0.03	0.07	0.26	− 0.14	0.19
NSA: composite score [% AL]	0.17	0.39	0.02	− 0.05	0.26	0.33	0.05	0.20

*p < .05; **p < .01; ***p < .001. AL: arm length, LSI: limb symmetry index; NSA: Non-stroke arm; SA: stroke arm; YBT-UQ: Y Balance Test-Upper Quarter; YoT: years of training

## Discussion

We investigated side differences between the stroke arm and non-stroke arm in upper quarter mobility/stability and their association with serve velocity in tennis players with different levels of training experience. Two novel results emerged: First, YBT-UQ side difference (i.e., composite score) and performance (i.e., medial reach) was larger in players with the least training experience (i.e., less than 2 years of training). Second, YBT-UQ performances (i.e., medial reach and composite score) but not side differences were significantly positively correlated with flat and slice serve velocity.

The first finding is contrary to our hypothesis stating that side differences would increase with advancing level of training experience. An increase in inter-limb asymmetry with increasing years of training has so far only been reported for the lower extremities [2] and thus cannot be transferred to the upper limbs. The detection of larger side differences in the composite score among players with the least versus highest training experience argues against the notion of a ‘functional specialisation’ due to long-term motor practice [1]. One reason could be that functional specialisation only occurs in sport-specific test conditions but not in non-specific ones [1]. In addition, the larger side differences in players with the least training experience indicate an increased injury risk [5]. Thus, injury prevention programs should be implemented in the tennis training routine, especially for less experienced players. On the other hand, the lower side differences for players with the highest experience level suggest that they may be more engaged in extensive fitness routines aimed at reducing such asymmetries, which may partly explain the lack of increased asymmetries [13].

The YBT-UQ performance (i.e., medial reach) was also greater in players with the least training experience when compared with the other two groups. This finding is in contrast to previous studies [14, 15] that reported better YBT-UQ performance with increasing competition level. For example, Bullock et al. [14] investigated high school and collegiate swimmers and found significantly better values for the medial reach direction in favour of the latter group of swimmers. The significantly greater reach distance in medial direction for players with the least training experience suggests a better direction-specific upper quarter mobility/stability compared to the other two groups of players.

The second finding is also contrary with our hypothesis stating that YBT-UQ side differences would be negatively associated with serve velocity. Meanwhile, our finding of positive correlations between YBT-UQ performance and serve velocity highlights that players with large medial reach values achieve higher serve velocities during flat and slice tennis serves, indicative of better upper quarter neuromuscular control. These correlations were observed when considering at all players, and the less experienced players in particular. This suggests that the level of upper quarter mobility/stability explains a greater proportion of variance with respect to serve velocity in players with a low compared to high level of training experience. From a practitioner’s perspective, it can be deduced that training-induced gains in upper quarter mobility/stability can be transferred to improvements in serve velocity, particularly in less experienced players.

## Conclusion

We determined whether side differences in upper quarter mobility/stability differ between tennis players with diverging levels of training experience and if they are related with serve velocity. Our results highlight that YBT-UQ side differences (i.e., composite score) and performances (i.e., medial reach) were largest in players with the lowest level of training experience (i.e., < 2 years). We further found that YBT-UQ performances (i.e., medial reach and composite score) but not side differences were significantly positively correlated with flat and slice serve velocity, particularly in less experienced players. These findings suggest meaningful inter-limb asymmetry in upper quarter mobility/stability in less experienced players, indicating an increased injury risk but no relationship with tennis-specific performance (i.e., serve velocity).

### Limitations

- Advanced players (ITN ≤ 4) were examined, which limits the transfer of findings to players with a lower skill level (i.e., intermediate or recreational).
- Assessment of side differences in upper quarter mobility/stability was restricted to a frequently used field test (i.e., YBT-UQ), which does not allow statements about other instrumented measures (e.g., shoulder strength or range of motion).
- The YBT-UQ represents a closed kinetic chain test and thus, our results cannot transfer to open chain assessments.
- The number of attempts to reach five successful strokes varied between the players.

## Electronic supplementary material

Below is the link to the electronic supplementary material.

Supplementary Material 1: Additional file 1 Participant performing the (A) medial, (B) inferolateral, and (C) superolateral reach direction of the Y Balance Test-Upper Quarter

## Data Availability

The data generated and analysed during the present study are not publicly available due to ethical restrictions but are available from the corresponding author upon reasonable request.

## Abbreviations

AL: Arm length
ANOVA: Analysis of variance
IL: Inferolateral
ITN: International Tennis Number
LSI: Limb symmetry index
MD: Medial
NSA: Non-stroke arm
SA: Stroke arm
SL: Superolateral
YBT-UQ: Y Balance Test-Upper Quarter
YoT: Years of training

## References

[1] Paillard T. Plasticity of the postural function to sport and/or motor experience. Neurosci Biobehav Rev. 2017;72:129–52. 10.1016/j.neubiorev.2016.11.015. 27894829 10.1016/j.neubiorev.2016.11.015

[2] Bishop C, Turner A, Read P. Effects of inter-limb asymmetries on physical and sports performance: a systematic review. J Sports Sci. 2018;36:1135–44. 10.1080/02640414.2017.1361894. 28767317 10.1080/02640414.2017.1361894

[3] Gonzalo-Skok O, Serna J, Rhea MR, Marín PJ. Relationships between functional movement tests and performance tests in young elite male basketball players. Int J Sports Phys Ther. 2015;10:628–38. 26491613 PMC4595916

[4] Madruga-Parera M, Romero-Rodríguez D, Bishop C, Beltran-Valls MR, Latinjak AT, Beato M, Fort-Vanmeerhaeghe A. Effects of maturation on lower limb neuromuscular asymmetries in elite youth tennis players. Sports (Basel). 2019. 10.3390/sports7050106. 31071938 10.3390/sports7050106 PMC6572117

[5] Teyhen DS, Shaffer SW, Goffar SL, Kiesel K, Butler RJ, Rhon DI, Plisky PJ. Identification of risk factors prospectively associated with musculoskeletal injury in a warrior athlete population. Sports Health. 2020;12:564–72. 10.1177/1941738120902991. 32134698 10.1177/1941738120902991 PMC7785899

[6] Swann C, Moran A, Piggott D. Defining elite athletes: issues in the study of expert performance in sport psychology. Psychol Sport Exerc. 2015;16:3–14. 10.1016/j.psychsport.2014.07.004.

[7] Teyhen DS, Riebel MA, McArthur DR, Savini M, Jones MJ, Goffar SL, et al. Normative data and the influence of age and gender on power, balance, flexibility, and functional movement in healthy service members. Mil Med. 2014;179:413–20. 10.7205/MILMED-D-13-00362. 24690966 10.7205/MILMED-D-13-00362

[8] Fernandez-Fernandez J, Nakamura FY, Moreno-Perez V, Lopez-Valenciano A, Del Coso J, Gallo-Salazar C, et al. Age and sex-related upper body performance differences in competitive young tennis players. PLoS ONE. 2019;14:e0221761. 10.1371/journal.pone.0221761. 31479492 10.1371/journal.pone.0221761 PMC6719856

[9] Dražen Harasin D, Dizdar G, Marković. High reliability of tests of maximum throwing performance. J Hum Mov Stud. 2007.

[10] Filipa A, Byrnes R, Paterno MV, Myer GD, Hewett TE. Neuromuscular training improves performance on the star excursion balance test in young female athletes. J Orthop Sports Phys Ther. 2010;40:551–8. 10.2519/jospt.2010.3325. 20710094 10.2519/jospt.2010.3325 PMC3439814

[11] Gokeler A, Welling W, Benjaminse A, Lemmink K, Seil R, Zaffagnini S. A critical analysis of limb symmetry indices of hop tests in athletes after anterior cruciate ligament reconstruction: a case control study. Orthop Traumatol Surg Res. 2017;103:947–51. 10.1016/j.otsr.2017.02.015. 28428033 10.1016/j.otsr.2017.02.015

[12] Taylor R. Interpretation of the correlation coefficient: a Basic Review. J Diagn Med Sonogr. 1990;6:35–9. 10.1177/875647939000600106.

[13] Fernandez-Fernandez J, Sanz-Rivas D, Mendez-Villanueva A. A review of the activity profile and physiological demands of tennis match play. Strength Conditioning J. 2009;31:15–26. 10.1519/SSC.0b013e3181ada1cb. 10.1519/JSC.0b013e318194208a 19197208

[14] Bullock GS, Brookreson N, Knab AM, Butler RJ. Examining fundamental movement competency and closed-chain upper-extremity dynamic balance in swimmers. J Strength Cond Res. 2017;31:1544–51. 10.1519/JSC.0000000000001627. 28538303 10.1519/JSC.0000000000001627

[15] Krysak S, Harnish CR, Plisky PJ, Knab AM, Bullock GS. Fundamental movement and dynamic balance disparities among varying skill levels in golfers. Int J Sports Phys Ther. 2019;14:537–45. 31440406 PMC6670059

