# Supplementary figures and images for: Side differences in upper quarter mobility/stability are not related to serve velocity in tennis players with different levels of training experience
Source: BMC Res Notes. 2024 Sep 26;17:275. doi: 10.1186/s13104-024-06944-z (PMC11430754; doi:10.1186/s13104-024-06944-z)

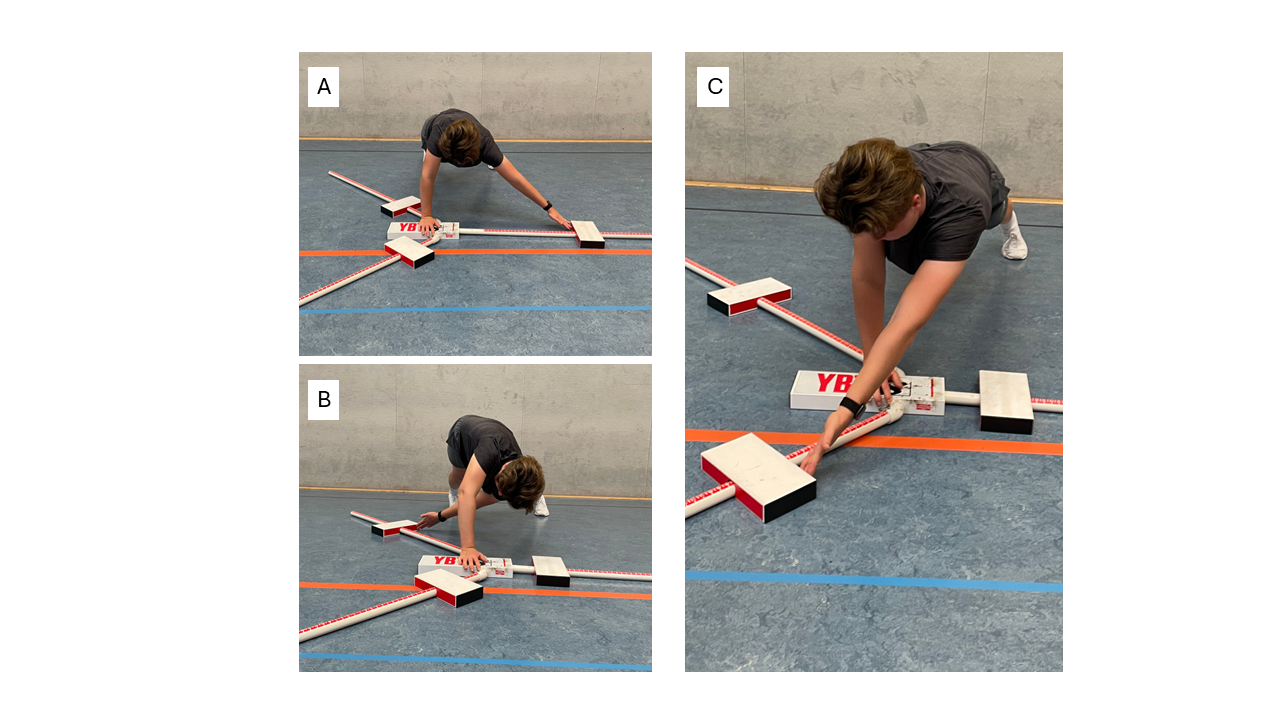

Supplement: Supplementary file 1 — Supplementary Material 1: Additional file 1 Participant performing the (A) medial, (B) inferolateral, and (C) superolateral reach direction of the Y Balance Test-Upper Quarter [file 13104_2024_6944_MOESM1_ESM.tiff]
